# Preoperative Predicting the WHO/ISUP Nuclear Grade of Clear Cell Renal Cell Carcinoma by Computed Tomography-Based Radiomics Features

**DOI:** 10.3390/jpm11010008

**Published:** 2020-12-23

**Authors:** Claudia-Gabriela Moldovanu, Bianca Boca, Andrei Lebovici, Attila Tamas-Szora, Diana Sorina Feier, Nicolae Crisan, Iulia Andras, Mircea Marian Buruian

**Affiliations:** 1 Department of Radiology and Medical Imaging, Faculty of Medicine, George Emil Palade University of Medicine, Pharmacy, Science and Technology of Târgu Mureș, 540139 Târgu Mureș, Romania; moldovanu_claudia@yahoo.com (C.-G.M.); mircea.buruian@umfst.ro (M.M.B.); 2 Department of Radiology, Emergency Clinical County Hospital of Cluj-Napoca, 400006 Cluj-Napoca, Romania; diana.feier@umfcluj.ro; 3 Department of Radiology, Faculty of Medicine, Iuliu Hațieganu University of Medicine and Pharmacy, 400012 Cluj-Napoca, Romania; 4 Department of Radiology, Clinical Municipal Hospital, 400139 Cluj-Napoca, Romania; attitamas@yahoo.com; 5 Department of Urology, Faculty of Medicine, Iuliu Hațieganu University of Medicine and Pharmacy, 400012 Cluj-Napoca, Romania; drnicolaecrisan@gmail.com (N.C.); dr.iuliaandras@gmail.com (I.A.); 6 Department of Radiology, Emergency Clinical County Hospital Târgu Mureș, 540136 Târgu Mureș, Romania

**Keywords:** clear cell renal cell carcinoma, radiomics, WHO/ISUP nuclear grade, multiphasic multidetector computed tomography

## Abstract

Nuclear grade is important for treatment selection and prognosis in patients with clear cell renal cell carcinoma (ccRCC). This study aimed to determine the ability of preoperative four-phase multiphasic multidetector computed tomography (MDCT)-based radiomics features to predict the WHO/ISUP nuclear grade. In all 102 patients with histologically confirmed ccRCC, the training set (*n* = 62) and validation set (*n* = 40) were randomly assigned. In both datasets, patients were categorized according to the WHO/ISUP grading system into low-grade ccRCC (grades 1 and 2) and high-grade ccRCC (grades 3 and 4). The feature selection process consisted of three steps, including least absolute shrinkage and selection operator (LASSO) regression analysis, and the radiomics scores were developed using 48 radiomics features (10 in the unenhanced phase, 17 in the corticomedullary (CM) phase, 14 in the nephrographic (NP) phase, and 7 in the excretory phase). The radiomics score (Rad-Score) derived from the CM phase achieved the best predictive ability, with a sensitivity, specificity, and an area under the curve (AUC) of 90.91%, 95.00%, and 0.97 in the training set. In the validation set, the Rad-Score derived from the NP phase achieved the best predictive ability, with a sensitivity, specificity, and an AUC of 72.73%, 85.30%, and 0.84. We constructed a complex model, adding the radiomics score for each of the phases to the clinicoradiological characteristics, and found significantly better performance in the discrimination of the nuclear grades of ccRCCs in all MDCT phases. The highest AUC of 0.99 (95% CI, 0.92–1.00, *p* < 0.0001) was demonstrated for the CM phase. Our results showed that the MDCT radiomics features may play a role as potential imaging biomarkers to preoperatively predict the WHO/ISUP grade of ccRCCs.

## 1. Introduction

Clear cell renal cell carcinoma (ccRCC) encompasses around 70% of all renal cell carcinomas, making it the most common pathological subtype [1,2]. It has the worst prognosis of all types of RCC, and its biological aggressiveness significantly changes the prognosis [3].

Tumor grading is among the most important prognostic factors as an independent predictor of cancer-specific survival for ccRCC stages [4]. The World Health Organization/International Society of Urological Pathology (WHO/ISUP) grading system for ccRCC [5] has improved interobserver reproducibility compared to the former Fuhrman grading system, being easier to apply and more clinically relevant. This four-grade system is based primarily on nucleolar prominence assessed to determine grades 1–3. Grade 4 is defined by the presence of highly atypical “pleomorphic” cells and/or sarcomatoid or rhabdoid morphology. Grades 1–2 are classified as low grades, and grades 3–4 are classified as high grades. Patients with low-grade ccRCC may be candidates for less invasive procedures, such as nephron-saving surgery, radiofrequency ablation, or active surveillance, whereas radical interventions are acceptable in patients with high-grade ccRCC [6].

Percutaneous renal mass biopsy is an accurate procedure that can identify the histology of the lesions [7]. Due to the heterogeneity of ccRCCs, the accuracy of tumor grading through biopsy is controversial, as the biopsy shows some discrepancies of the resection sample for grading systems. Some studies focusing on renal tumor biopsies and tumor grading [8,9,10,11] have reported that biopsies usually underestimate the final grade and less often overestimate the final grade. The percentage of accurate biopsy grading was reported between 43% and 75%, and the percentage of differentiation between low and high grade was reported between 64% and 87%. Moreover, different parts of a tumor have distinct molecular characteristics and such differences change over time. Thus, optimal characterization of tumor grading by percutaneous biopsy cannot be obtained properly because it is not possible to biopsy each part of a tumor at different times [12,13].

The field of medical and biological image analysis has recently grown exponentially, and a new method called radiomics has been developed [14,15,16]. Radiomics is a promising technique that extracts and analyzes large numbers of imaging features to provide more information than only human imaging evaluation can offer. This method uses high-throughput extraction of large numbers of quantitative radiomics features obtained from medical images using advanced mathematical algorithms to determine tumor phenotypes [17,18,19]. Thus, the heterogeneity of the entire tumor volume is assessed compared to biopsies that assess the heterogeneity in a small portion of the tumor and at a single anatomical site [20,21,22,23,24]. Several previous studies [25,26,27,28,29,30,31,32] have shown that radiomics features based on multiphasic multidetector computed tomography (MDCT) images perform efficiently in differentiating between high-/low-grade ccRCC tumors. Given these promising results, we assume that MDCT-based radiomics features may play a feasible role in predicting high-/low-grade ccRCCs. This study aims to evaluate if radiomics features extracted from a four-phase MDCT study may be helpful to preoperatively differentiate the WHO/ISUP nuclear grades of ccRCCs.

## 2. Materials and Methods

The ethical approval for this retrospective study was obtained from the Institutional Review Board of Clinical Municipal Hospital of Cluj-Napoca (Approval Code: No. 15/2020; Approval Date: 11 June 2020). No formal written consent was required for this study.

### 2.1. Study Population

We performed a retrospective analysis of the medical database for patients with pathologically proven ccRCC from January 2018 to February 2020. The inclusion criteria were as follows: patients with four-phase MDCT scan before surgery; WHO/ISUP nuclear grades, which were available from the pathology reports. The exclusion criteria were: significant artifacts on images (motion or metal artifacts), previous tumor treatment, and patients with cystic lesions. Our study comprised 102 patients (mean age: 61.92 ± 13.03), which were divided into two groups: the training set (62 patients) and the validation set (40 patients).

### 2.2. Image Acquisition

MDCT scans were performed with a 64-row scanner (Somatom Sensation, Siemens, Erlangen, Germany) using: a 120 kV variable tube current (variable setting from 200 to 400 mAs based on patient size); section collimation, 0.6 mm; table feed, 5 mm/s; slice thickness, 3.0 mm; and a pitch of 1. Nonionic contrast material was injected via an antecubital vein at a rate of 3.0 mL/s using a CT-compatible power injector with a total volume of 80–150 mL. A region of interest (ROI) in the thoracoabdominal aorta junction was placed, with a trigger set to begin at 150 HU. The renal mass protocol consisted of a four-phase study: an unenhanced (UN) scan followed by contrast-enhanced acquisitions during the corticomedullary (CM, 30 s delay), nephrographic (NP, 90 s delay), and excretory (EX, 8 min delay) phases.

### 2.3. Histopathological Assessment of Nuclear Grade

WHO/ISUP nuclear grades were obtained from the pathology reports of the histopathological examination. The samples were obtained from the partial nephrectomy of 22 patients, total nephrectomy of 13 patients, and radical nephrectomy of 67 patients. All tumors were divided into low-grade ccRCC (WHO/ISUP grades 1 and 2) and high-grade ccRCC (WHO/ISUP grades 3 and 4).

### 2.4. Tumor Segmentation, Preprocessing, and Radiomics Feature Extraction

**Figure 1:**
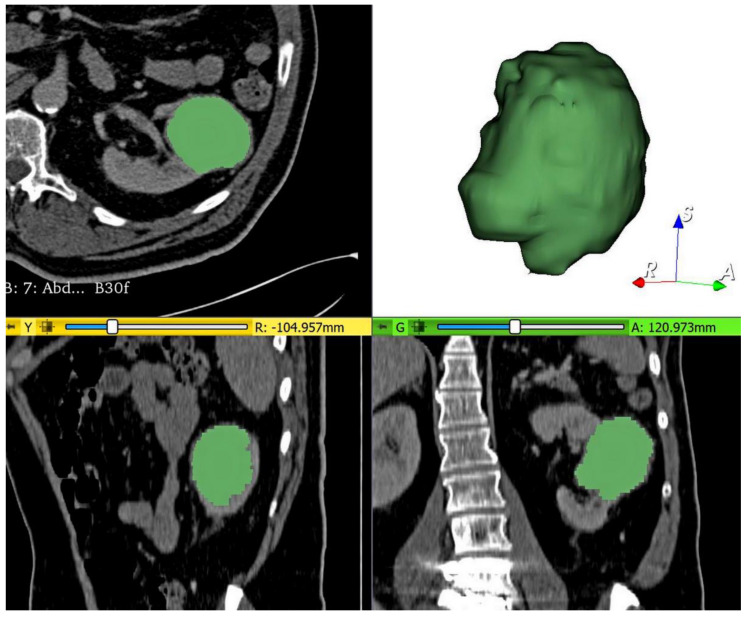
Example of volume of interest (VOI) segmentation in the nephrographic (NP) phase of a pathologically proven clear cell renal cell carcinoma (ccRCC).

From the pictured archiving and communication system (PACS, Carestream, Canada), all MDCT acquisitions were exported and transferred to a workstation to be segmented using the open-source 3D Slicer software, version 4.10.2 (www.slicer.org). For each renal mass, the volume of interest (VOI) segmentation was manually and slightly delineated slice by slice by a radiology resident (Claudia-Gabriela Moldovanu), in accordance with a senior radiologist with 9 years of experience in urogenital imaging (Attila Tamas-Szora) to ensure the accuracy of the tumor boundaries. The two radiologists were blinded to the pathological results. To minimize the partial volume effect from surrounding structures, the segmentations were carefully delineated, reducing the size of the tumors by 1 mm from the current visible edge. The nephrographic phase was used for segmentation because it provides an adequate delimitation between the tumor and uninvolved adjacent parenchyma (Figure 1).

Prior to radiomics features extraction, the images of each patient were preprocessed: first, the images and VOIs were resampled to an isotropic voxel size of 1 × 1 × 1 mm^3^ using B-Spline interpolation; then, normalization of the images was performed by centering in the chosen place by division through standard deviation; finally, image discretization was performed of the gray level by a fixed bin width of 25 in the histogram.

A total of 4184 radiomics features of the four-phase MDCT study per patient (1046 features per phase) were extracted from the VOIs and divided into four groups: (1) image intensity (first-order statistics features); (2) shape and size-based features; (3) second-order statistics features (textural features); and (4) higher-order statistical features (obtained after applying filters and mathematical transforms to the images). We used Laplacian transforms of Gaussian-filter- and wavelet-transformed images. The Laplacian of Gaussian (LoG) filter was used with values of 2 mm, 4 mm, and 6 mm, representing fine, medium, and coarse patterns, respectively. Wavelet-based texture features were generated using eight different frequency band combinations, applying either a high- or low-pass filter in each of the three dimensions including high–high–high, high–high–low, high–low–low, high–low–high, low–high–low, low–high–high, low–low–high, and low–low–low. Radiomics features were extracted from images with and without preprocessing filters from all four MDCT phases separately. PyRadiomics version 2.1.2. was used for both preprocessing and feature extraction.

### 2.5. Reliability Validation of Texture Features

According to previously published guidelines [33,34,35], the reproducibility of texture features was calculated using the interclass correlation coefficient (ICC) of the radiomics features. Another radiologist (Andrei Lebovici, with 8 years of experience in urogenital imaging) independently resegmented all renal masses and extracted radiomics features, also blinded by the pathological results. Thus, for each extracted texture features, the ICC was calculated. For the feature selection process, the features with an ICC value of ≥0.75 were included, indicating excellent reproducibility, resulting in a total of 3429 features (826 in the UN phase, 861 in the CM phase, 864 in the NP phase, and 878 in the EX phase).

### 2.6. Statistical Analysis

Statistical analysis was performed using SPSS Statistics software for Windows, version 18.0 (SPSS Inc., Chicago, IL, USA) and R software version 3.6.3 using the “glmnet” package. The Mann–Whitney U-test was used for univariate analysis to identify the features with a significant difference between low/high-grade ccRCC groups. The Benjamini–Hochberg (BH) correction method was applied to control the false discovery rate in multiple hypothesis testing. BH-adjusted *p*-values < 0.05 were considered significant. Spearman’s correlation coefficient was used to assess the correlation between all radiomics features. This was performed between any two features, and when the Spearman coefficient was > 0.9/< −0.9, the feature with the higher *p*-value in the univariate analysis was eliminated. For standard comparison and mitigating the effects of the data splitting, the radiomics scores were built using the least absolute shrinkage and selection operator (LASSO) performed by 10-fold cross-validation. The radiomics score (Rad-Score) was computed for each MDCT phase of each patient through a linear combination of features weighted by their LASSO coefficients. To evaluate the predictive performance of the radiomics score for the differentiating ability of low/high-grade ccRCC in the training and validation sets, the area under the receiver operating characteristic (ROC) curve (AUC) was used, and *p* < 0.05 was considered statistically significant. Multivariate analysis using binary logistic regression (enter method) was conducted to detect independent predictors of the WHO/ISUP nuclear grade of ccRCCs, including the clinicoradiological characteristics and radiomics score as independent variables.

## 3. Results

### 3.1. Patients Characteristics

A total of 102 patients (mean age: 61.92 ± 13.03) were included in this study, divided into training sets and validation sets based on the random split method. Thus, 62 patients constituted the training set (40 men, 22 women; mean age: 61.09 ± 12.64), whereas 40 patients constituted the validation set (27 men, 13 women; mean age: 63.2 ± 13.66). In the training set, 40 patients were classified according to the WHO/ISUP grading system as low-grade ccRCC, and the remaining 22 patients were classified as high-grade ccRCC. The validation set comprised 29 patients with low-grade ccRCC and 11 patients with high-grade ccRCC. The baseline characteristics of training and validation sets are provided in Table 1.

No significant difference in the gender of the patients, N stage, and intratumoral necrosis between low- and high-grade ccRCC in both the training and validation sets was observed. However, significant differences were observed in the ages of the patients, tumor size, tumor stage, vein thrombosis, perinephric fat invasion, intratumoral neovascularity, and intratumoral hemorrhage in the training set. These results are partially confirmed in the validation set, where tumor size, tumor stage, and vein thrombosis were the only significantly different characteristics.

### 3.2. Feature Selection and Radiomics Score Building: Training Set

Feature selection and radiomics score building were separately performed on each MDCT phase of each patient. According to the standard of ICC ≥ 0.75 in the inter-reader agreement evaluation, 826 radiomics features from the UN phase, 861 features from the CM phase, 864 features from the NP phase, and 878 features from the EX phase were highly reproducible and selected for further analysis.

To develop the radiomics signature, univariate analysis was performed to assess the potential of the radiomics features to differentiate between the low- and high-grade ccRCC groups. Excluding those with an adjusted *p*-value > 0.05, the number of features was further decreased to 1241 features (228 in the UN phase, 387 in the CM phase, 340 in the NP phase, and 286 in the EX phase). These features were included in the further selection process.

After applying the Spearman correlation analysis, these features were secondly reduced to 302 potential predictors (46 in the UN phase, 110 in the CM phase, 85 in the NP phase, and 61 in the EX phase). Furthermore, the LASSO binary logistic regression algorithm was used to reduce the dimensionality of the above high-dimensional features; thus, the best features were selected based on the optimal λ parameters. Forty-eight radiomics features with nonzero coefficients were then selected to construct the radiomics scores across all MDCT phases (10 in the UN phase, 17 in the CM phase, 14 in the NP phase, and 7 in the EX phase). Most of the features included in the radiomics scores were obtained from filtered images using LoG and wavelet-transformed filters, being mainly texture and first-order features (Table 2).

A significant difference in the radiomics scores between low- and high-grade ccRCCs in all MDCT phases, with patients from the second group having higher values (Table 3), was observed. Rad-Score was calculated according to the following formula:

$$\mathrm{Rad}-\mathrm{Score}\;=\;\sum_{c=0}^{a}Cc\;*\;{\mathrm{Xc}}_{\;}{+}_{\;}b$$

where *a* is the number of radiomics features with nonzero coefficients for each MDCT phase (10 for the UN phase, 17 for the CM phase, 14 for the NP phase, and 7 for the EX phase), *Cc* is the coefficient of the cth feature, Xc the cth feature, and b the intercept.

### 3.3. Performance of the Radiomics Scores: Training Set

**Figure 2:**
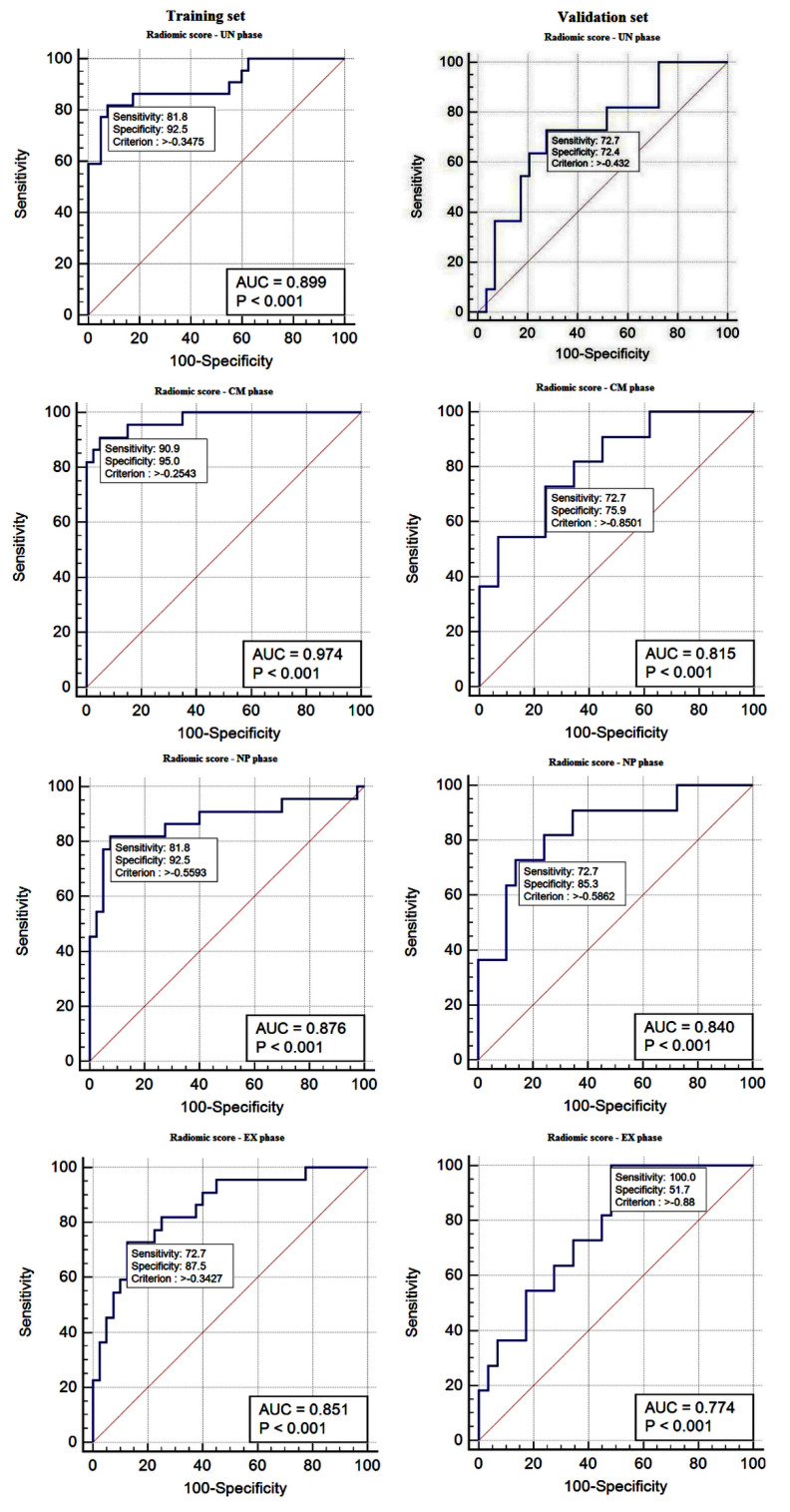
ROC curves of radiomics scores of all MDCT phases in the training and validation sets. ROC, receiver operating characteristic, AUC area under the curve.

To compare the detection performance, the Rad-Scores were validated in terms of ROC curve and AUC in the training set (Figure 2). Sensitivity, specificity, positive predictive value (PPV), and negative predictive value (NPV) were also calculated. The radiomics scores showed a favorable predictive efficacy for differentiating low- from high-grade ccRCC based on each phase of the MDCT protocol. The results are summarized in Table 4. In the training set, the Rad-Scores derived from the UN and CM phases achieved the best predictive ability, with a sensitivity, specificity, and an AUC of 81.82%, 92.50%, and 0.89 in the UN phase and 90.91%, 95.00%, and 0.97 in the CM phase.

Using the variables with a significant difference among low- and high-grade ccRCCs in the training set (including age, tumor size, vein thrombosis, perinephric invasion, tumor stage (2–4), intratumoral neovascularity, and hemorrhage), we conducted a multivariate logistic regression analysis to develop a clinicoradiological model for the preoperative prediction of the WHO/ISUP nuclear grade of ccRCCs (Table 5). Further, we constructed four complex models, adding the radiomics score of each phase to the clinicoradiological model (Table 6).

**Figure 3:**
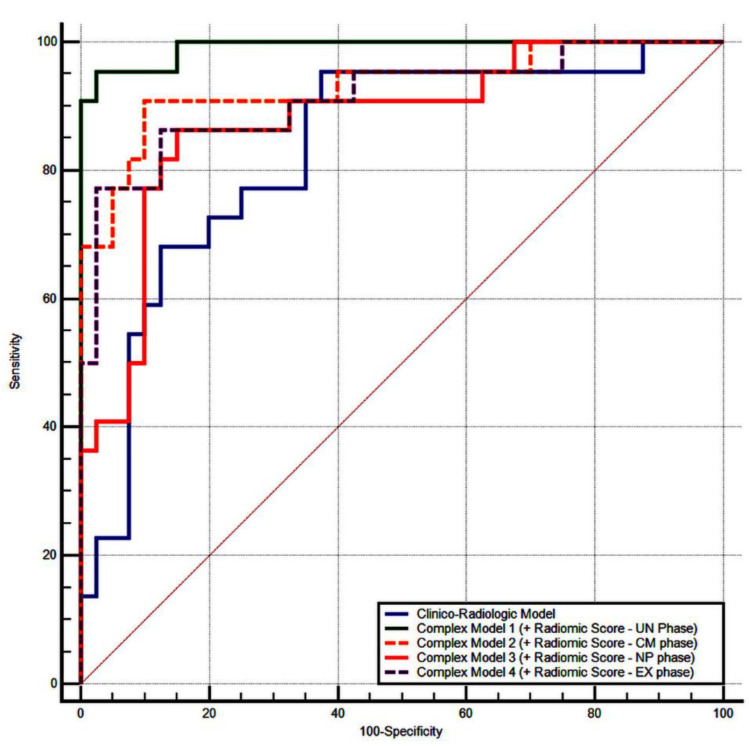
ROC curves of the clincoradiological model and complex models to categorize the nuclear grades of ccRCC.

The ability of the clincoradiological model and the complex model to categorize nuclear grades was evaluated by the AUC of the ROC curves (Figure 3). The clincoradiological model showed a high performance in the discrimination of low- and high-grade ccRCCs with an AUC of 0.83 (95% CI, 0.71–0.91, *p* < 0.0001). We found that the addition of radiomics score to the clincoradiological characteristics improved their performance in all MDCT phases: AUC = 0.93 (95% CI, 0.84–0.98, *p* < 0.0001) vs. AUC = 0.89 (95% CI, 0.79–0.96, *p* < 0.0001) in the UN phase, AUC = 0.99 (95% CI, 0.92–1.00, *p* < 0.0001) vs. AUC = 0.97 (95% CI, 0.89–0.99, *p* < 0.0001) in the CM phase, AUC = 0.91 (95% CI, 0.81–0.97, *p* < 0.0001) vs. AUC = 0.87 (95% CI, 0.76–0.94, *p* < 0.0001) in the NP phase, AUC = 0.87 (95% CI, 0.77–0.94, *p* < 0.0001) vs. AUC = 0.85 (95% CI, 0.73–0.92, *p* < 0.0001) in the EX phase.

### 3.4. Validation of the Radiomics Score

The performance of the Rad-Scores for the discrimination of low- and high-grade ccRCCs was confirmed in the validation set in each MDCT phase of each patient (Table 3). ROC curve analysis was conducted, and the AUC, sensitivity, specificity, PPV, and NPV for the determined cut-off values were calculated. The results are presented in Figure 2 and Table 4. Compared with the training set, in the validation set, the Rad-Scores derived from the CM and NP phases achieved the best predictive ability, with a sensitivity, specificity, and an AUC of 72.73%, 75.90%, and 0.81 in the CM phase and 72.73%, 85.30%, and 0.84 in the NP phase.

## 4. Discussion

In this study, we evaluated if radiomics features extracted from a four-phase MDCT study may be helpful to preoperatively differentiate the WHO/ISUP nuclear grades of ccRCC. In the era of personalized medicine, radiomics features, along with metabolic, histopathologic, and genetic datasets, may be useful to improve patient management, a biomarker that could be useful in tumor characterization, treatment selection, and prognosis [36,37,38,39]. Many radiomics features have proven to be useful in differentiating between early- and advanced-stage diseases of various types of cancers [40,41,42,43]. In recent years, concerning renal imaging, little research [25,26,27,28,29,30,31,32] has investigated the radiomics potential based on MDCT to predict the ccRCC nuclear grade. Regarding the histologic tumor grading system, the majority of studies used the Fuhrman classification system as a pathological reference. Although the Fuhrman and WHO/ISUP grading systems are linboth used in current medical practice for ccRCC grading, some studies [44,45,46,47] have reported that the Fuhrman grading system has poor interobserver reproducibility compared to the new WHO/ISUP grading system.

In recent years, the WHO/ISUP grading system has been accepted in current medical practice, replacing the former Fuhrman grading system. To the best of our knowledge, there are only a few published papers that have studied radiomics features based on MDCT for predicting the ccRCC WHO/ISUP nuclear grade [29,48,49,50,51]. However, no previous work used parameters extracted from a four-phase MDCT study to develop the prediction model, as our study does.

Our results show that our constructed MDCT-based radiomics scores using a four-phase protocol achieved a considerably promising performance in differentiating high- from low-grade ccRCCs. The Rad-Scores derived from the UN and CM phases achieved the best predictive ability in the training set. However, in the validation set, the Rad-Scores from the CM and NP phases achieved the best predictive ability. We found that the best predictive ability with an AUC of 0.94 was for the CM phase in the training dataset and 0.84 was for the NP phase in the validation datasets. This diversity illuminates that the CM and NP phases are valuable and necessary for ccRCC grading. Our results are in concordance with the results of previous studies on ccRCC grading using texture analysis or machine learning (ML), which reported an accuracy between 0.78 and 0.82 and an AUC between 0.71 and 0.98 [29,48,49].

Our feature selection results showed that the first-order features and second-order statistics features were significantly associated with the WHO/ISUP grade. In building our radiomics scores, most of the features included were obtained from filtered images, especially from wavelet-transformed filters. Shu et al. [50] used two predictive models constructed by radiomics features extracted from the nephrographic and medullary phases and reported no significant difference in the AUC between them to differentiate low- from high-grade ccRCC. Conversely, they showed that the combined model of radiomics features from two certain phases had the highest differential diagnostic efficiency (AUC: 0.82 (95% CI: 0.76–0.86). A recent study [51] showed that the value of the NP phase is limited in predicting the ISUP grade. This may be due to two reasons: firstly, regarding tumor delineation, Sun et al. used a single-slice approach (largest cross-section diameter of the tumor) and did not perform data analysis of the entire tumor VOI. Although VOIs segmentations are time-consuming processes, we believe that the single-slice approach does not fully reflect the heterogeneity of the tumor, and the information obtained from the VOI might be more reliable for the characterization of the tumor. Secondly, their features extraction algorithm is different; they extracted the radiomics features from original and wavelet-filtered images, without the use of LoG filters. It is known that filtered-based images can limit the impact of technical noise [52]. More and more studies are using them, but a current technical standardization regarding their use has not yet been established [53].

MRI-derived ADC values are useful in characterizing tumor activity [54]. Some studies [55,56] that evaluated the utility of ADC to differentiate low- from high-grade ccRCC reported that MRI has a favorable predictive accuracy in detecting high-grade ccRCC (AUC = 0.80). With all its advantages, MRI is not as widely used as MDCT for the analysis of renal masses, being used only in selected cases. Cui et al. [48] used MRI- and CT-based radiomics models to differentiate low- from high-grade ccRCCs, and then the authors compared their performance. They reported that radiomics models based on a three-phase MDCT performed better than the radiomics model based on a single-phase MDCT, with an ACC ranging from 77 to 79% in internal validation and 61 to 69% in external validation.

Similarly, the radiomics model based on all-sequence MRI was also superior to the radiomics model based on single-sequence MRI, with an ACC ranging from 71 to 73% in internal validation and 64 to 74% in external validation. When comparing the performance between MDCT and MRI, they found that the MRI-based radiomics model had performed better than the MDCT-based radiomics model for diagnosing low-grade ccRCC and showed a similar ability for diagnosing high-grade ccRCC.

Regarding the statistical approach, our study included one classification method: the binary logistic regression method. This algorithm is used to predict the probability of the class of a categorical dependent variable [57]. Several studies [58,59] have assessed the performance of quantitative MDCT texture analysis combined with different machine-learning-based classifiers to discriminate low- from high-grade ccRCC. It was observed that the highest predictive performance was obtained by the support vector machine classifier. However, these results were obtained for the Fuhrman grading system of ccRCC. Despite differences in the procedure followed, we believe that all studies support each other with the same conclusion that MDCT-based radiomics features may be a promising noninvasive method in predicting preoperative ccRCC grades.

In this study, the radiomics scores combined with the clinicoradiological characteristics showed a high performance in the discrimination of ccRCC grades. Two characteristics (age and tumor stage) were consistent with previous studies [29,56]. Li et al. proved a correlation between radiological characteristics and the ccRCC nuclear grade [28]. Shape, margin, and necrosis may be independent predictors of high-grade ccRCC, whereas a regular shape can often be seen in low-grade ccRCC lesions [28]. Another paper [60] demonstrated statistically significant differences in WHO/ISUP grading and pT staging between ccRCCs. In addition, they found that coagulative necrosis often occurs in high-grade and high-stage tumors.

This study may have important practical implications. The new WHO/ISUP grading system is a prognostic factor for ccRCCs. ccRCC grades were strongly related to patient outcomes and tumor biological behavior [61,62]. If low-grade tumors can be identified preoperatively, the treatment would consist of less invasive procedures. Moreover, partial nephrectomy can preserve partial renal function, thus reducing overall mortality and the incidence of cardiovascular disease [63]. Therefore, medical images can become a valuable source of information, and radiomics features may be used as a noninvasive method for characterizing and classifying lesions. However, further larger prospective studies to validate the performance of our proposed radiomics model in differentiating high from low-grade ccRCC are necessary for the future.

The present study has some limitations. (1) It was a single-center retrospective study with a small sample size of patients. (2) The statistical approach included one classification method, the binary logistic regression method, and advanced classifiers may offer better prediction performance. (3) External validation in more centers with more samples size is needed to overcome these limitations and validate the results in order to improve generalization and evaluate the potential for clinical translation of our radiomics models. (4) Volume effect interference cannot be completely avoided due to the fact that the tumor boundary was manually drawn. (5) The four-phase MDCT renal mass protocol involves a high dose of radiation to the patient and should be performed where it is necessary to discriminate the lesions before treatment selection.

## 5. Conclusions

Although there are limitations with regard to sample size, we have shown that radiomics features extracted from the four-phase MDCT study may play a role as a potential imaging biomarker to predict preoperatively the WHO/ISUP grade of ccRCCs, helping urologists to better stratify patients and choose the best treatment.

## Figures and Tables

**Table 1:** Demographic and clinicoradiological characteristics of the study population. * *p* < 0.05 was considered statistically significant.

	Training Set			Validation Set		
Characteristic	Low Grade	High Grade	*p*-Value	Low Grade	High Grade	*p*-Value
Number	40	22		29	11	
Age (years)	58.2 ± 12.92	66.36 ± 10.45	0.009 *	61.89 ± 13.30	66.63 ± 14.66	0.36
Gender			0.91			1.00
Male	26 (65%)	14 (35%)		20 (74.1%)	7 (25.9%)	
Female	14 (63.6%)	8 (36.4%)		9 (69.2%)	4 (30.8%)	
Tumor size (mm)	46.65 ± 28.53	73.22 ± 26.25	0.001 *	53.17 ± 22.68	79.45 ± 25.15	0.008 *
Tumor stage (n)			0.001 *			0.01 *
1	30 (85.7%)	5 (14.3%)		18 (94.7%)	1 (5.3%)	
2	3 (50%)	3 (50%)		6 (100%)	-	
3	7 (35%)	13 (65%)		5 (35.7)	9 (64.3%)	
4	-	1 (100%)		-	1 (100%)	
Vein thrombosis			0.02 *			0.009 *
No	33 (73.3%)	12 (26.6%)		22 (88%)	3 (12%)	
Yes	7 (41.1%)	10 (58.8%)		7 (46.6%)	8 (53.3%)	
Tumor necrosis			0.47			1.00
No	7 (77.7%)	2 (22.2%)		2 (66.6%)	1 (33.3%)	
Yes	33 (60%)	22 (40%)		27 (72.9%)	10 (27.0%)	
Perinephritic invasion			0.009 *			0.49
No	34 (73.9%)	12 (26.0%)		12 (80%)	3 (20%)	
Yes	6 (37.5%)	10 (62.5%)		17 (68%)	8 (32%)	
Intratumoral neovascularity			0.003 *			0.29
No	30 (78.9%)	8 (21.0%)		11 (85.6%)	2 (15.3%)	
Yes	10 (41.6%)	14 (58.3%)		18 (66.6%)	9 (33.3%)	
Hemorrhage			0.01 *			0.48
No	32 (74.4%)	11 (25.5%)		16 (80%)	4 (20%)	
Yes	8 (42.1%)	11 (57.8%)		13 (65%)	7 (35%)	
Lymphadenopathy			0.05			0.12
No	35 (71.4%)	14 (28.5%)		27 (77.1%)	8 (22.8%)	
Yes	5 (38.4%)	8 (61.5%)		2 (40%)	3 (60%)	

**Table 2:** List of selected radiomics features and their coefficients for calculating the radiomics score.

Radiomic Group	Radiomic Feature	Associated Filter	Coefficient
**UN phase**			
	Intercept		−0.872
Texture feature	JointAverage	LoG filter (2 mm)	0.409
Texture feature	SizeZoneNonUniformity	LoG filter (2 mm)	0.010
Texture feature	DependenceVariance	LoG filter (4 mm)	0.362
First-order	Minimum	LoG filter (4 mm)	−0.296
Texture feature	LongRunEmphasis	LoG filter (4 mm)	0.477
Texture feature	SmallAreaHighGrayLevelEmphasis	LoG filter (4 mm)	0.091
Texture feature	LargeAreaLowGrayLevelEmphasis	Wavelet-LHL	0.039
Texture feature	LongRunLowGrayLevelEmphasis	Wavelet-LLH	−0.431
Texture feature	SmallAreaLowGrayLevelEmphasis	Wavelet-LLH	−0.349
Texture feature	LongRunLowGrayLevelEmphasis	Wavelet-HHL	0.343
**CM phase**			
	Intercept		−1.184
Texture feature	SmallAreaLowGrayLevelEmphasis	Original	−0.387
First-order	Skewness	LoG filter (2 mm)	−0.311
First-order	Minimum	LoG filter (2 mm)	−0.052
First-order	10Percentile	LoG filter (2 mm)	0.303
Texture feature	LowGrayLevelEmphasis	LoG filter (4 mm)	−0.373
Texture feature	LongRunHighGrayLevelEmphasis	Wavelet-HLL	0.306
Texture feature	LowGrayLevelZoneEmphasis	Wavelet-HLL	−0.076
Texture feature	Imc2	Wavelet-LHL	0.797
First-order	Mean	Wavelet-LHL	0.516
Texture feature	GrayLevelNonUniformity	Wavelet-LHL	−0.153
Texture feature	SmallAreaEmphasis	Wavelet-LHL	0.823
Texture feature	LongRunLowGrayLevelEmphasis	Wavelet-LLH	−0.429
First-order	Maximum	Wavelet-HLH	0.583
Texture feature	GrayLevelVariance	Wavelet-HHL	0.084
First-order	Entropy	Wavelet-HHL	0.049
Texture feature	RunVariance	Wavelet-HHL	0.064
Texture feature	ShortRunLowGrayLevelEmphasis	Wavelet-LLL	−0.379
**NP phase**			
	Intercept		−0.765
Texture feature	HighGrayLevelRunEmphasis	Original	0.325
Texture feature	ShortRunHighGrayLevelEmphasis	LoG filter (6 mm)	−0.087
Texture feature	Imc2	Wavelet-HLL	0.191
Texture feature	ShortRunHighGrayLevelEmphasis	Wavelet-HLL	0.225
Texture feature	Contrast	Wavelet-LHL	0.192
Texture feature	SmallAreaHighGrayLevelEmphasis	Wavelet-LHL	0.353
Texture feature	ZoneEntropy	Wavelet-LHL	0.049
First-order	Entropy	Wavelet-LHH	0.070
Texture feature	DependenceNonUniformityNormalized	Wavelet-LLH	0.013
Texture feature	SumEntropy	Wavelet-HLH	−0.190
Texture feature	Imc2	Wavelet-HLH	0.331
Texture feature	GrayLevelVariance	Wavelet-HHL	0.019
Texture feature	Idn	Wavelet-LLL	0.223
Texture feature	SmallAreaLowGrayLevelEmphasis	Wavelet-LLL	−0.189
**EX phase**			
	Intercept		−0.653
Texture feature	DependenceVariance	LoG filter (4 mm)	−0.234
First-order	Kurtosis	LoG filter (4 mm)	0.139
Texture feature	RunVariance	LoG filter (4 mm)	0.163
Texture feature	SizeZoneNonUniformity	LoG filter (4 mm)	0.032
Texture feature	DependenceNonUniformityNormalized	Wavelet-HLL	0.165
Texture feature	SmallDependenceLowGrayLevelEmphasis	Wavelet-HLL	−0.046
Texture feature	SmallAreaHighGrayLevelEmphasis	Wavelet-LHL	0.028

**Table 3:** Difference of the radiomics score (Rad-Score) between low- and high-grade ccRCC in the training and validation sets.

WHO/ISUP Nuclear Grades	Radiomic Score Mean ± SD	*p*-Value	MDCT Phase
**Training set**			
Low grade (n = 40)	−1.68 ± 1.16	*p* < 0.001	UN
	−2.50 ± 1.95	*p* < 0.001	CM
	−1.26 ± 0.68	*p* < 0.001	NP
	−0.92 ± 0.53	*p* < 0.001	EX
High grade (n = 22)	0.60 ± 1.34	*p* < 0.001	UN
	1.21 ± 1.29	*p* < 0.001	CM
	0.15 ± 1.18	*p* < 0.001	NP
	−0.16 ± 0.51	*p* < 0.001	EX
**Validation set**			
Low grade (n = 29)	−1.18 ± 1.70	*p* = 0.051	UN
	−2.21 ± 2.42	*p* < 0.001	CM
	−1.12 ± 0.72	*p* = 0.001	NP
	−0.80 ± 0.62	*p* = 0.009	EX
High grade (n = 11)	−0.03 ± 1.32	*p* = 0.051	UN
	1.53 ± 3.43	*p* < 0.001	CM
	0.19 ± 1.56	*p* = 0.001	NP
	−0.24 ± 0.45	*p* = 0.009	EX

**Table 4:** Radiomic score performance in the training and validation sets in all MDCT phases.

Variable	AUC (95% CI)	Se (95% CI)	Sp (95% CI)	PPV (95% CI)	NPV (95% CI)	Cut-Off Value	*p*-Value
**Training set**							
Radiomic score: UN phase	0.89 (0.796–0.961)	81.82 (59.7–94.8)	92.50 (79.6–98.4)	85.7 (63.7–97.0)	90.2 (76.9–97.3)	−0.34	<0.001
Radiomic score: CM phase	0.97 (0.89–0.99)	90.91 (70.8–98.9)	95.00 (83.1–99.4)	90.9 (70.8–98.9)	95.0 (83.1–99.4)	−0.25	<0.001
Radiomic score: NP phase	0.87 (0.76–0.94)	81.82 (59.7–94.8)	92.50 (79.6–98.4)	85.7 (63.7–97.0)	90.2 (76.9–97.3)	−0.55	<0.001
Radiomic score: EX phase	0.85 (0.73–0.92)	72.73 (49.8–89.3)	87.50 (73.2–95.8)	76.2 (52.8–91.8)	85.4 (70.8–94.4)	−0.34	<0.001
**Validation set**							
Radiomic score: UN phase	0.72 (0.56–0.85)	72.73 (39.0–94.0)	72.41 (52.8–87.3)	50.0 (24.7–75.3)	87.5 (67.6–97.3)	−0.43	0.0157
Radiomic score: CM phase	0.81 (0.66–0.92)	72.73 (39.0–94.0)	75.90 (56.5–89.7)	53.3 (26.6–78.7)	88.0 (68.8–97.5)	−0.85	<0.001
Radiomic score: NP phase	0.84 (0.69–0.93)	72.73 (39.0–94.0)	85.30 (68.3–96.1)	66.7 (34.9–90.1)	89.3 (71.8–97.7)	−0.58	<0.001
Radiomic score: EX phase	0.77 (0.61–0.89)	100.0 (71.5–100.0)	51.72 (32.5–70.6)	44.0 (24.4–65.1)	100.0 (78.2–100.0)	−0.88	<0.001

**Table 5:** Multivariate logistic regression analysis for the preoperatively prediction of the WHO/ISUP nuclear grade of ccRCCs: clinicoradiological model.

Variable	Coefficient	Std. Error	*p*-Value	Odds Ratio (OR)
Age (years)	0.05	0.03	0.10	1.05
Tumor size (mm)	0.006	0.01	0.74	1.00
Vein thrombosis: positive	−1.32	1.22	0.27	0.26
Perinephric invasion: positive	1.60	1.39	0.25	4.98
Tumor stage (2, 3, or 4)	2.09	1.07	0.05	8.13
Intratumoral neovascularity: positive	1.04	0.99	0.29	2.85
Hemorrhage: positive	−1.81	1.55	0.24	0.16
Constant	−5.55			

**Table 6:** Multivariate logistic regression analysis for the preoperative prediction of the WHO/ISUP nuclear grade of ccRCCs: complex model.

Variable	Coefficient	Std. Error	*p*-Value	Odds Ratio (OR)
**UN phase**				
Age (years)	0.03	0.05	0.4997	1.03
Tumor size (mm)	−0.02	0.02	0.4156	0.97
Vein thrombosis: positive	1.64	1.81	0.3653	5.16
Perinephric invasion: positive	−0.17	1.73	0.9176	0.83
Tumor stage (2, 3, or 4)	0.99	1.42	0.4860	2.70
Intratumoral neovascularity: positive	0.07	1.17	0.9485	1.07
Hemorrhage: positive	−1.75	1.87	0.34	0.17
Radiomic score: UN phase	1.83	0.59	0.0021	6.27
Constant	−0.90			
**CM phase**				
Age (years)	0.12	0.09	0.1772	1.13
Tumor size (mm)	−0.08	0.07	0.2576	0.91
Vein thrombosis: positive	1.13	16.78	0.9460	3.11
Perinephric invasion: positive	2.76	17.36	0.8735	15.86
Tumor stage (2, 3, or 4)	3.62	3.38	0.2837	37.59
Intratumoral neovascularity: positive	−2.36	2.85	0.4075	3.11
Hemorrhage: positive	−4.07	17.52	0.8161	0.01
Radiomic score: CM phase	4.92	2.37	0.0384	137.75
Constant	−1.50			
**NP phase**				
Age (years)	0.11	0.05	0.03	1.12
Tumor size (mm)	−0.03	0.02	0.2121	0.96
Vein thrombosis: positive	−3.15	1.68	0.0610	0.04
Perinephric invasion: positive	3.88	2.23	0.0827	48.76
Tumor stage (2, 3, or 4)	2.19	1.50	0.1449	8.98
Intratumoral neovascularity: positive	0.34	1.24	0.7830	1.41
Hemorrhage: positive				
Radiomic score: NP phase	2.78	0.91	0.0023	16.17
Constant	−4.64			
**EX phase**				
Age (years)	0.05	0.04	0.2408	1.05
Tumor size (mm)	−0.05	0.03	0.0879	0.94
Vein thrombosis: positive	−0.24	1.42	0.8639	0.78
Perinephric invasion: positive	1.01	1.57	0.5184	2.77
Tumor stage (2, 3, or 4)	1.87	1.19	0.1170	6.49
Intratumoral neovascularity: positive	0.09	1.07	0.9263	1.10
Hemorrhage: positive	−1.20	1.71	0.4832	0.29
Radiomic score: EX phase	4.64	1.75	0.0081	103.88
Constant	1.40			
